# Assessing Quality of Program Environments for Children and Youth with Autism: Autism Program Environment Rating Scale (APERS)

**DOI:** 10.1007/s10803-017-3379-7

**Published:** 2017-11-20

**Authors:** Samuel L. Odom, Ann Cox, John Sideris, Kara A. Hume, Susan Hedges, Suzanne Kucharczyk, Evelyn Shaw, Brian A. Boyd, Stephanie Reszka, Jennifer Neitzel

**Affiliations:** 1 Frank Porter Graham Child Development Institute, University of North Carolina at Chapel Hill, CB 8180, Chapel Hill, NC 27599-8180 USA; 2 Department of Special Education, University of Arkansas, Fayetteville, AR USA; 3 Department of Allied Health Sciences, University of North Carolina at Chapel Hill, Chapel Hill, NC 27599 USA

**Keywords:** Autism, Quality, Rating scale, Psychometrics

## Abstract

The purpose of this study was to examine the psychometric properties of the *Autism Program Environment Rating Scale (APERS)*, an instrument designed to assess quality of program environments for students with autism spectrum disorder. Data sets from two samples of public school programs that provided services to children and youth with autism spectrum disorder were utilized. Cronbach alpha analyses indicated high coefficients of internal consistency for the total *APERS* and moderate levels for item domains for the first data set, which was replicated with the second data set. A factor analysis of the first data set indicated that all domain scores loaded on one main factor, in alignment with the conceptual model, with this finding being replicated in the second data set. Also, the *APERS* was sensitive to changes resulting from a professional development program designed to promote program quality.

## Introduction

Autism spectrum disorder (ASD) has markedly increased in prevalence in the last two decades. Once thought to be an extremely low prevalence disorder (i.e., 2 in 10,000 children identified in the early 1980s), the current prevalence rate is 1 in 68 (Baio 2014; Christensen et al. 2016). In the United States, there has been a parallel increase in the number of students with ASD enrolled in special education programs [i.e., a 350% increase 2004–2013 according to Office of Special Education Programs (2015)]. With this increase has come the need to provide appropriate and high quality educational programs, and in fact, parents of children with ASD have brought legal actions against school systems for not providing such programs (Zirkel 2011). When school systems lose these law suits, it is in part because they are not able to document the quality of the programs they provide (Yell et al. 2003). The purpose of this paper is to describe the development of an assessment of program quality for students with ASD, called the *Autism Program Environment Rating Scale* (*APERS*) and its psychometric characteristics. After a brief review of previous research on assessment of program quality for students with ASD, the *APERS* instrument development process and history will be described, followed by a report of its reliability and validity. The paper will conclude with a discussion of the instrument’s utility and current use in research, program evaluation, and professional development.

For this study, “program” will refer to educational services operating in public schools for children and youth with ASD. The *Individuals with Disabilities Education Improvement Act* (IDEIA 2004) specifies that for children with disabilities between the ages of 3 and 22, public schools have to provide free and appropriate educational services in the least restrictive environment. The law provides a number of specific details about these requirements (e.g., each student must have an individualized education plan; transition program has to start at 16). Environment refers to the physical, instructional, and social features of classrooms and other locations at the school. The professional and research literature, as well as IDEA, specifies the quality of program services, which will be discussed subsequently.

The assessment of the quality of educational program environments has been the strongest in early childhood education. The *Early Childhood Environment Rating Scale* (Harms et al. 2014) has the longest history, with the *Classroom Assessment Scoring System* (Pianta et al. 2008) also well established. Both have sound psychometric qualities and are used extensively in state and national evaluations of early childhood education programs. The *Inclusive Classroom Profile* (*ICP*, Soukakou 2016) has extended assessment of early childhood education environments to program features that support children with disabilities. The *ICP* also has sound psychometric qualities (Soukakou 2012).

Assessment of program quality for individuals with ASD has been more limited, although some rating scales have been developed. The *Environmental Rating Scale* (*ERS*) (Van Bourgondien et al. 1998) is a staff-administered rating scale designed to measure residential environments for adolescents and adults with ASD. The authors have published evidence of reliability and construct validity, although only 52 adolescents and adults with autism participated in the study. In a revision of the *ERS*, Hubel, Hagell, and Sivberg (2008) developed a questionnaire version of the scale, the *ERS-Q*, which could be completed by program staff. They found high internal consistency and substantial concurrent validity between the two scales, although again the number of participants in the study was quite low (n = 18). Also, this scale focused on out-of-school/residential settings for adolescents and young adults rather than school-based programs across the grade range.

To examine learning environments for school-age children and youth with ASD, researchers have created several scales. For schools in Belgium, Renty and Roeyers (2005) developed a questionnaire to examine special education and inclusive school programs. The questionnaire was completed by school staff and gathered information about services available, modification of the school environment, staff knowledge of ASD, and parent involvement. The authors did not, however, provide information about the psychometric qualities of their questionnaire and there have been no reports of its use in the United States. A team from the state of New York (Crimmins et al. 2001) developed the *Autism Program Quality Indicators*. This scale assessed the quality of environments for children with ASD and contained sections addressing both program (e.g., personnel, curriculum, family involvement) and student considerations (e.g., assessment, transitions) (Librera et al. 2004). However, no information is available about its reliability and validity. To measure features of quality in programs for learners with ASD, the Professional Development in Autism program, funded from 2002 to 2007, developed the *PDA Program Assessment* (Professional Development in Autism, n.d.), which was a checklist containing items representing eight domains. Again, no evidence of reliability or validity has been reported for this measure. Similarly, several state committees (Colorado Department of Education 2016; Kansas State Department of Education, Special Education Services 2013; Librera et al. 2004) have developed assessments of quality programs, often intended for teacher self-study of their programs, with none providing psychometric information.

A team from the National Professional Development Center on Autism Spectrum Disorders (NPDC 2011) developed the *APERS*. The NPDC was a multi-site center with collaborators from universities in North Carolina, Wisconsin, and California. This center, funded by the U. S. Department of Education, Office of Special Education Programs, was charged to design a professional development program that states would use to promote service-providers’ implementation of evidence-based practices in educational programs for children and youth with ASD. States applied to work with the NPDC to create their professional development program, and NPDC worked with 12 states over a 4-year period. A state-level team made up of administrators and special education leaders in the state identified six classroom programs that would initially participate in the professional development program and eventually serve as models for other programs.

**Fig. 1:**
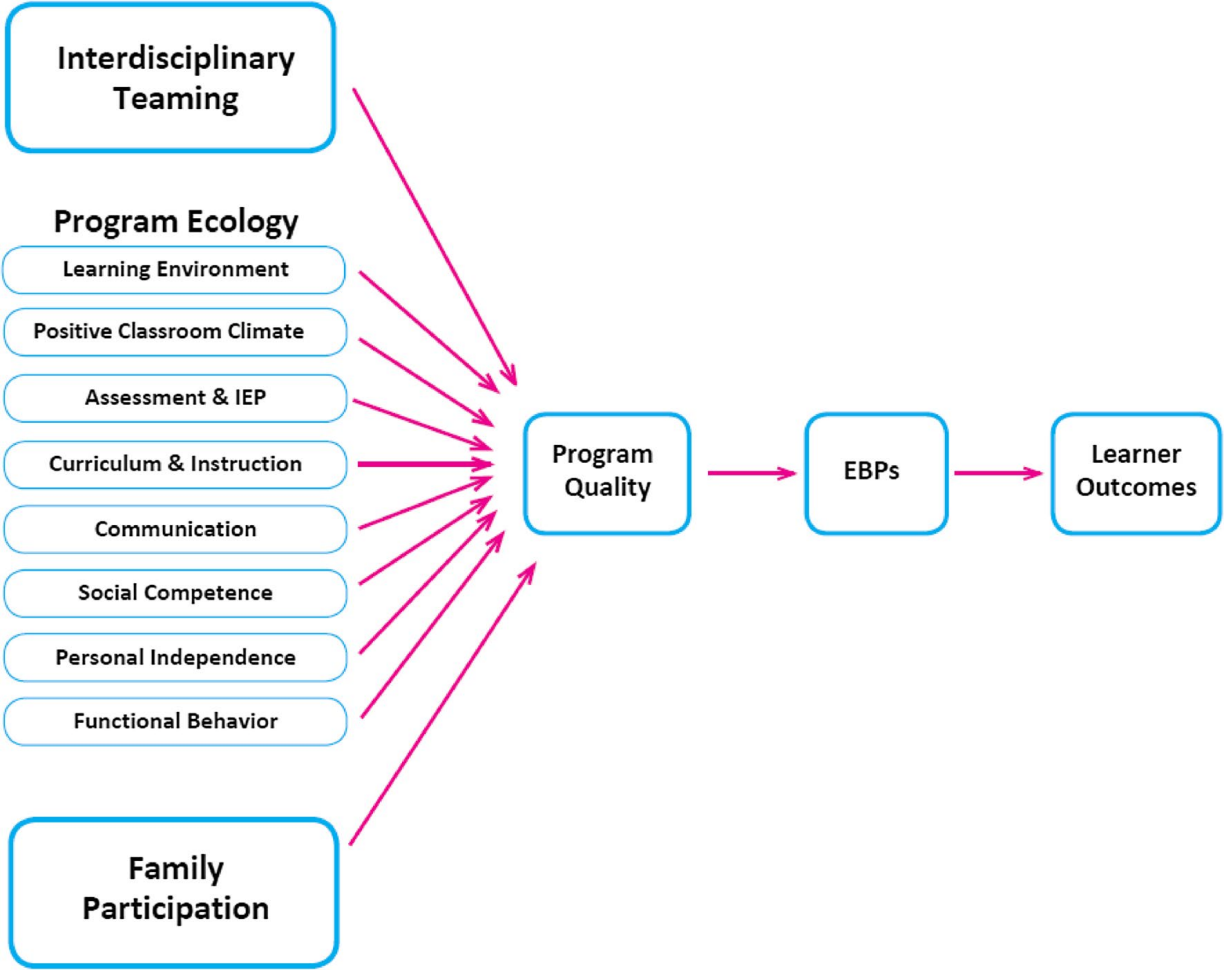
Conceptual model of program quality for students with ASD

The *APERS* was an integral part of the NPDC. The operating assumption of NPDC was that the quality of a program serves as the “platform” on which EBPs are implemented, with practitioners’ ease of implementation being directly associated with the level of program quality (Odom et al. 2013). This assumption is similar to the concept in clinical psychology of high quality therapy services having “common factors” (Deegear and Lawson 2003) that serve as the context for specific evidence-based therapies design to address special conditions such as depression or anxiety (Lampropoulos 2000). Recent evidence supports this model in psychotherapy (Wampold 2015). The reason for developing the *APERS* rather than using other previously developed scales was that the previous scales (a) did not cover all the program areas and features the NPDC team deemed necessary to assess program quality (see Fig. 1), (b) did not have the level of detail sufficient to provide comprehensive formative feedback to programs (i.e., to improve the educational “common elements” serving as the foundation for implementation of EBPs), and/or (c) did not have evidence of reliability and validity.

### Theoretical Rationale for APERS

The theoretical foundation for the *APERS* is based on a traditional Bronfenbrenner (1979) view of ecological systems, in this case at the micro- and meso-system levels, and their influence on individual development and learning. In addition, the *APERS* draws from the logic of IDEIA, and the assumption that certain features of programs are necessary and important for learners with disabilities. A set of program features exists within classrooms or more broadly school programs, which are the primary educational microsystem(s) in which the learner participates. These include structure and organization, social climate of the classes, assessment, and “content” related instructional or intervention practices that map onto the challenges most common for students with ASD (e.g., communication, social competence, behavior). In addition, there are meso-system influences; that is, influences from outside the immediate classroom/program. These are construed as the interdisciplinary teaming process and family participation. These features (i.e., defined in the *APERS* as domains) contribute to a cumulative program experience for individual learners with ASD, which we propose is the “quality” of the program (see Fig. 1.). Table 1 contains a brief description of each domain.

**Table 1:** Description of *APERS* domain content

APERS domains	Description of content
Learning environments	Safety, organization, materials, visual schedules, transitions
Positive learning climate	Staff-student interactions, staff behaviors
Assessment and IEP development	Assessing student progress, assessment process, IEP goals, transition planning
Curriculum and instruction	Classroom instruction, focus on IEP goals, opportunity to generalize, prompting, accommodations
Communication	Planning for communication, communication rich environment, individualized communication instruction, responsiveness to student, communication systems
Social competence	Arranging opportunities, teaching and modeling, personal hygiene and relationships, social skills training, peer social networks
Personal independence	Self-advocate for accommodations, self-management, choices available
Functional behavior	Proactive strategies, behavioral assessment, data collection, teaming
Family involvement	Teaming, communication, parent teacher meetings
Teaming	Team membership, team meetings, decision making

### Development of the APERS

The development of the *APERS*, which began in 2007, has been systematic and iterative. The selection of items and the format for the *APERS* began with a review of the literature on assessment of program and learning environments for children and youth who are typically developing and for individuals with ASD. During the instrument development process, NPDC staff selected and modified items from other scales (e.g., the PDA checklist, the ECERS) or created applicable items, sorted items into categories that reflected important features of learning environment and programs, shared analyses with other members of the team, revised items based on feedback, and assembled items into domains. Team members then wrote item anchors, shared anchors with other team members for review, and revised item anchors based on feedback.

The items and domains were assembled into the *APERS Preschool*/*Elementary (APERS PE) version*. This version was then shared with a team of experts and practitioners in middle school and high school programs for learners with ASD and modified to create the middle/high school version [*APERS Middle*/*High School (APERS MH)*]. Items were revised to represent the different quality features of middle/high school programs. Initially one domain was added to include a focus on transition; however, the transition items were subsequently embedded within the other domains in the 2013 version of the *APERS*.

NPDC research team members then pilot tested the *APERS* in preschool, elementary, middle, and high school programs for learners with ASD in public schools in one school district. Pilot test information was used to revise observation and data collection procedures as well as individual items. Both versions of the *APERS* were then distributed to the research staff at the three sites of the NPDC (NC, WI, CA). Staff administered the *APERS* in the fall of 2008 and spring of 2009 in early childhood, elementary, and middle/high school programs. These staff members provided detailed feedback about items and data collection procedures, which were incorporated into the 2011 versions of the *APERS*.

In 2013, research team members with the Center on Secondary Education for Students with Autism Spectrum Disorders (CSESA) made slight modifications to the middle/high school version of the *APERS*. The CSESA is a research and development center funded by the U. S. Department of Education-Institute of Education Sciences to establish and evaluate a comprehensive treatment program for adolescents with ASD in high schools (see reference blinded, to be included in final manuscript). Like the NPDC model, CSESA staff initially collected *APERS* information and provided feedback to school staff in order to build the foundation of quality of subsequent implementation of evidence-based program features. The CSESA model was more comprehensive than the NPDC model in that it was applicable to all programs for students with ASD operating in a specific school (i.e., NPDC typically only operated with the self-contained or inclusive program in a school). The slight modifications were insertion of item notes for the coders to assist them in interpreting the scoring anchors and rubric as applicable for a broader school context. Items and items anchors from the earlier version were not changed, with one exception. The items from the transition domain in the NPDC version were integrated into content domains (e.g., Assessment, Teaming) in the CSESA version.

### Research Questions

The purpose of this paper is to examine the psychometric quality of the *APERS*. The specific questions addressed and hypotheses are:

1. What is the reliability of the *APERS*? The authors hypothesized that there would be a high level of internal consistency and moderate to strong inter-rater agreement.
2. What is the construct validity of the *APERS*? The authors had two hypotheses: (a) in a confirmatory factor analysis, a single factor reflecting quality would be detected and all *APERS* domains would load significantly on this factor (see Fig. 1), and (b) the *APERS* would be sensitive to changes resulting from a professional development program designed to improve program quality.

## Method

This study draws from two databases—the original data collection that occurred through the NPDC Project and the data collected through the CSESA project (reference blinded, to be included in final manuscript). The schools and participants will be described in different sections aligned with the analyses. The rationale for including two data sets was to allow replication across samples. Also, because of the nature of the CSESA study, additional research questions that were not possible with the NPDC sample (i.e., inter-rater agreement) could be addressed.

### Sample 1: NPDC

#### Instrument

**Fig. 2:**
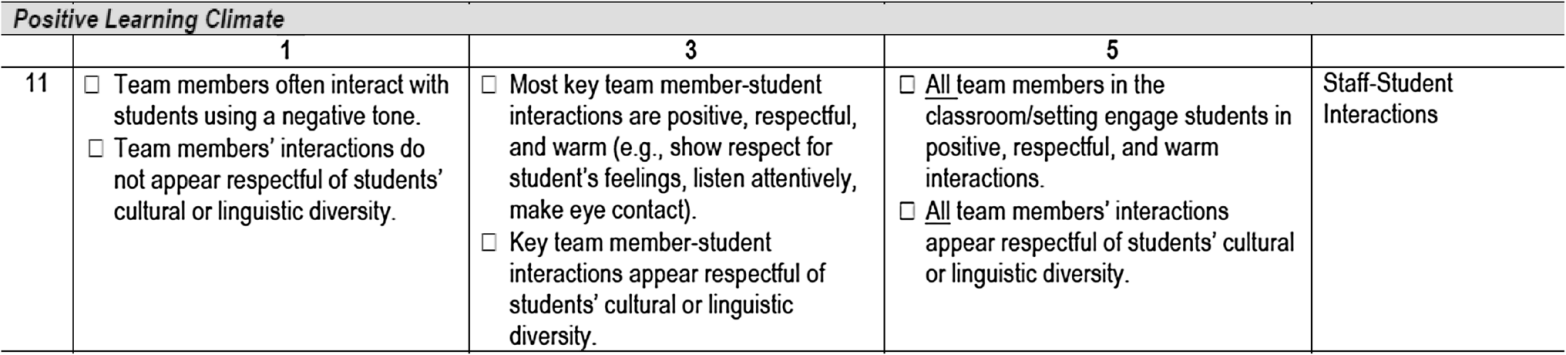
Example of scoring rubric for APERS

As noted, separate versions of the *APERS* were created to measure specific aspects of program quality for two age ranges: (a) the *APERS-PE* and (b) the *APERS-MHS*. The *APERS* items are organized on a five point rating continuum, with the 1 rating indicating poorest quality and 5 indicating high quality. An example of the rating rubric appears in Fig. 2. To score a 1, the rater codes any indicator in the 1 rating (i.e., 1 rating will occur regardless of any subsequent indicators scored for higher ratings). For a score of 2, at least one but not all of the 3 rating indicators is coded. For a score of 3, all 3 rating indicators are coded. For a score of 4, all 3 rating indicators are coded and at least one (but not all) 5 rating indicators is/are coded. For a score of 5, all 3 and all 5 rating indicators are coded. The coding format is computer-based. The rater codes the individual indicators that reflect quality on a specific item and the software program generates the score for the item and also tabulates domain (e.g., Assessment, Teaming, Families) and total mean item ratings.

To gather information for scoring the *APERS*, trained coders observed classes and contexts in programs for learners with ASD for 3–4 h that were distributed across 2 days. For items that were not easily observed on a short visit, coders interviewed the lead teacher, team members, and selected family members. They also reviewed students’ Individual Education Plans as well as any other relevant documentation (e.g., behavior intervention plans, individualized transition plans). For NPDC’s work, two children or youth with ASD were identified as “focal students”, in order to situate the observations in classes, school, or community contexts. The criteria for selecting the focal students were that they had an established educational diagnosis of ASD, as defined by their respective states, and that the school staff nominated them as being representative of the students receiving services in the program (e.g., if the program were self-contained and most students had intellectual disability and had limited communication skills, then the focal students had these characteristics).

The *APERS-PE* is designed to be used in programs serving children with ASD who are 3–10 years of age. The *APERS-PE* includes 64 items organized originally into 11 domains, although two previously separate learning environment domains were merged because of comparable content, resulting in 10 domains. Each of the domains are included in Fig. 1. The *APERS-MHS* was designed to be used in programs serving learners with ASD who are 11–22 years of age (or the end high school). The *APERS-MHS* includes 66 items and 11 domains that focus on all aspects of program quality (i.e., the one extra domain was transition, which was merged with other content domains for this analysis for comparability with the subsequent analysis to be described.).

#### Rater Training

Staff at each project site participated in a training regimen in which they reviewed items in each domain, discussed interpretation of the items, and practiced observation and item rating with feedback from the trainer, who was one of the original developers of the *APERS*. They then conducted a complete *APERS* assessment with a trained rater, examined agreement between ratings and reached consensus on items scored separately. If sufficient consensus occurred between coder in training and trained rater, they then conducted the *APERS* independently. Sufficient consensus was defined as agreeing within one point on the majority of items and agreeing on interpretation of items, with the trained coder, on items not within one point. Because of the national context, geographical disbursements of site, and resource limitations, inter-observer agreement was not collected for this sample.

#### Settings

NPDC program staff collected *APERS* in 72 school-based programs located in 11 states (CA, ID, IN, KY, MI, MN, NM, TX, VA, WI, and WY) in the Fall of the school year. Programs were located in preschool, elementary, middle, and high schools. Selection criteria were: (a) schools in which programs were located had to part of a local education agency (i.e., not charter or private schools); (b) schools had to have inclusive (i.e., defined by students with ASD spending 80% or more of their time in general education classes) and/or self-contained programs for students with ASD; and (c) programs could not be in self-contained schools that only enrolled students with disabilities. The state leadership team identified the specific programs based on geographic distribution around the state (e.g., a program in the north, central, and south parts of the state in north–south oriented state) as well as distribution across preschool/elementary, middle/high school programs. The exception to this process was if the state leadership identified a specific need for the state (e.g., one state team specified middle school as a particular need area), in which case it was given priority. The distribution of grade range and inclusive/ self-contained classes appears in Table 2. Although the states were nationally distributed and the students with ASD in programs were from racial/ethnically diverse families, specific data on the racial/ethnic diversity of the students in the autism programs were not available.

**Table 2:** Distribution of NPDC programs by grade-level and type

Program (level, type)	Preschool	Elementary	Middle	High	Total
Inclusive	9	20	5	8	42
Self-contained	5	9	10	6	30
Total	14	29	15	14	72

### Study 2: CSESA Sample

The CSESA sample was drawn from a randomized clinical trial (RCT) study that took place in 60 high schools located in central North Carolina, central and northern Wisconsin, and southern California. Selection criteria for these schools were the same as stated previously for the NPDC sample. The student participants in the larger RCT study were racially/ethnically diverse, with 43% of the sample classified (by parents) as either African-American or other designated racial group or White-Hispanic. An integral part of the CSESA model is using the *APERS*, as happened with NPDC, to assess program quality and share with the school staff to create a school-improvement plan.

#### Instrument

The CSESA staff modified the *APERS-MHS* in 2013 based on an item analysis, which resulted in the CSESA version of the *APERS-MHS* having 10 domains, and 65 items. Previously, the *APERS* had been designed to assess one program (e.g., a self-contained autism class OR an inclusive program) in a school. As noted previously, the CSESA project modified the instrument to reflect “whole school” quality (e.g. quality of both the self-contained and inclusive classes across the school contexts). As a result, CSESA staff observed in a wider variety of program locations in schools and interviewed a larger set of personnel than occurred for NPDC.

#### Settings

The CSESA staff, as noted, collected data for students in inclusive and self-contained special education programs separately within the same school in the Fall of the school year. School programs began in either the 9th or 10th grade, depending on the local education agency. Students with ASD in the inclusive programs usually graduate with their typically developing peers (i.e., around age 18), whereas students in the self-contained programs may stay until age 22. For the CSESA study, staff selected three students with ASD to situate observations in the school (i.e., at least one from self-contained, one from inclusive, and the third from either based on school context). All of these students met their state’s criteria for eligibility for special education services under the autism category. Like the NPDC sample, all schools were publically funded (i.e., no charter schools) and none were self-contained, special education schools that only had classes for students with disabilities.

#### Coder Training

Training for coders was conducted following the same process described previously for the NPDC sample. However, for the CSESA sample a trained reliability coder visited each site, simultaneous conducted observations, interviews, and record reviews, and independently scored the *APERS* for the school for approximately 20% of the sample.

#### APERS Data Collection

In the fall of the school year, CSESA staff collected the *APERS* data for 56 inclusive and 43 self-contained programs located in 60 high schools (high schools often had both inclusive and non-inclusive programs).

## Results

### Inter-rater Agreement

For the CSESA sample, two research staff simultaneously collected *APERS* information and completed *APERS* ratings in 21 programs. Inter-rater agreement at the item level was calculated in two ways: agreement within one rating point and exact agreement. The mean percentage agreement was 95.2 and 76.5% respectively. An ICC was also calculated, yielding a coefficient of .56, and a Pearson correlation based on the mean item rating for the total score by the two coders was .54 (p <. 01). Although we did not have sufficient numbers of ratings to conduct a traditional generalizability study, we did examine mean item ratings averaged across all schools for the two raters. The mean scores were 3.31 (SD = .55) and 3.33 (SD = 0.53) for the two raters. Item ratings were also examined at the individual item level and the difference between coders were calculated. For example, if one coder scored 3 and the other scored 5, the difference was 2; if they both scored the same rating, the difference was 0. The mean item rating difference on this five-point scale (i.e., calculated by subtracting the rating by one coder on an individual item from the rating by the second observer and dividing by the number of items) was .37 (SD = .35).

### Question 1: Reliability of the APERS

Data from the NPDC and CSESA projects allowed us to address the question of internal consistency. To examine the former, Cronbach alphas (Cronbach 1951, 1970) were computed for the total score and also for the individual domains of the NPDC and CSESA data sets. First, we computed the alphas for the Preschool-Elementary and Middle-High School forms of the *APERS*. As can be seen in Table 3, the alphas for both were quite high (.94–.96). The domain scores were lower but nearly all were above the .70 level. Second, to examine the *APERS* conducted for inclusive and non-inclusive programs, we combined the Preschool-Elementary and Middle-High School dataset, renumbering items so that the items for the two versions “matched up” (i.e., some items had slightly different numbers in the two formats and we matched equivalent items). We then conducted alphas for the inclusive and self-contained settings separately, again finding high alphas for the total (.94–.96) and lower alphas for the domains. For the CSESA data, we calculated alphas for the inclusive and self-contained programs separately. These findings replicate the results from the NPDC data set in that the total score alphas were high (.94 and .96) and domains were lower but generally in the 0.70–.80 range.

**Table 3:** Internal consistency by source, age group, and program type

	NPDC				CSESA	
	P/E	MHS	Incl	S/C	Incl	S/C
Total	.96	.96	.96	.94	.95	.96
Learning environment	.69	.71	.76	.73	.82	.81
Positive learning climate	.76	.78	.61	.77	.71	.68
Assessment/IEP development	.84	.87	.86	.86	.60	.76
Curriculum and instruction	.81	.77	.85	.84	.87	.89
Communication	.79	.92	.84	.73	.69	.74
Social	.72	.78	.73	.63	.70	.75
Personal independence and competence	.75	.78	.75	.76	.70	.75
Functional behavior	.85	.81	.76	.68	.81	.81
Family involvement	.88	.76	.78	.68	.74	.68
Teaming	.85	.71	.72	.74	.68	.60

### Question 2a: Construct Validity–Factor Structure

To test our proposed factor structure for the *APERS*, we conducted a confirmatory factor analysis (CFA) with the NPDC data set and then repeated the model with the CSESA data set. We based our analyses on the domain scores as the measurement variables. In the initial CFA  (see Table 4), we tested a one factor model where all domains were free to load on the latent variable. The analysis confirmed model fit for the single factor solution (RMSEA = .13, CFI = .90, and Chi square (35) = 74.63, p = .0001). The high RMSEA value is of concern as it is outside the commonly accepted guideline of less than .05 for good fit and .08 for acceptable fit. RMSEA is impacted by sample size and is positively biased (i.e., too large) when sample sizes are small (e.g., Rigdon 1996). Recent researchers have argued against its use for small samples or low degree-of-freedom models (Kenny, Kanistan, & McCoach, 2015). Rather than omitting RMSEA, we chose to relax our standard relative to common practice.

**Table 4:** Factor loadings for NPDC sample

*APERS* domains	One factor
Learning environment structure/schedule	0.61
Positive learning climate	0.63
Assessment/IEP development	0.72
Curriculum and instruction	0.87
Communication	0.67
Social	0.68
Personal independence and competence	0.79
Functional behavior (interfering and adaptive)	0.67
Family involvement	0.57
Teaming	0.61
Model fit	
Degrees of freedom	35
Chi square	149.81 p = .0000
RMSEA	.14
CFI	.87

We next applied the same structure to the CSESA data. In most of the CSESA schools, we observed two programs: one inclusive, the other self-contained (see Table 5). To account for possible school-level effects in addition to program-level effects in the factor structure, we first fit a multi-level CFA (MCFA) where we allowed for both within program and between program estimates for the factor loadings. The within program loadings are of particular interest, with the between program loadings included primarily to account for the nesting of programs within schools. Model fit for this was comparable to the fit for the NPDC CFA models: RMSEA = .11, CFI = .83, and Chi square (70) = 152.74, p = .0000. However, when the clustering is ignored, model fit is slightly improved (RMSEA = .10, CFI = .92, and Chi square (35) = 70.21, p = .0004) suggesting that the correction is unnecessary (for a more complete discussion of the determination of model selection in the presence of nested data, see Stapleton et al. 2016). The factor loadings for the unadjusted model are very similar to the loadings in the NPDC CFA.

**Table 5:** Factor loadings for CSESA sample

*APERS* domains	With clustering	Ignoring clustering
Learning environment structure/schedule	0.71	0.62
Positive learning climate	0.60	0.63
Assessment/IEP development	0.49	0.61
Curriculum and instruction	0.83	0.89
Communication	0.68	0.77
Social	0.26	0.61
Personal independence and competence	0.69	0.75
Functional behavior (interfering and adaptive)	0.72	0.63
Family involvement	0.40	0.50
Teaming	0.43	0.59
Model fit		
Degrees of freedom	70	35
Chi square	152.74 p = .0000	70.21 p = .0004
RMSEA	.11	.10
CFI	.83	.92

These analyses have been based on factor scores. Having selected a one factor solution and determined that entire sample is best conceived of representing a single population, we tested whether a simple sum score would be adequate compared to score based on the factor scoring. To do this, we refit the CFA with the constraint that all factor loadings were fixed to one. Model fit was very similar to the model with free factor loadings: RMSEA = .12, CFI = .84, and Chi square (44) = 84.74, p = .0000. This difference is not statistically significant [Chi square (9) = 14.52, p = .1048] and examination of the domain factor loadings on the total indicates that they are similar to each other. We conclude that a single sum score based on the *APERS* domains sufficiently captures the variance in the scale.

### Question 2b: Construct Validity–Sensitivity to Program Effects

Assessment instruments are sometimes used to assess the impact of reform or interventions to enhance quality. For those instruments, sensitivity to detecting changes that are predicted by a hypothesis can be viewed as a measure of construct validity (Guyatt et al. 1987; Nunnally and Bernstein 1994). For the NPDC project, staff conducted the *APERS* in the fall of the academic year and used those data to design and implement a plan with the school staff that would improve quality in the school. NPDC staff then conducted an *APERS* assessment again in the spring of the school year. The hypothesis was that *APERS* scores would increase. Univariate t tests were conducted to determine the difference between the pre and post *APERS* mean item ratings, which indicated a significant difference between the two time points for the total and all domain scores (all t > 4.38; all p < .001; all d > .50; see Table 6). These findings were consistent with the hypothesis and suggest sensitivity of the instrument to program effects. It is important to note that a subset of these data appeared in a previous article published by (Odom et al. 2013).

**Table 6:** Pre-Post changes on NPDC sample of *APERS*

	*Point estimates*						*t*	*d*
	*Pre*			*Post*				
	*N*	*Mean*	*Std*	*N*	*Mean*	*Std*		
Total	68	3.50	0.61	66	4.16	0.46	9.77***	1.08
Learning environment structure/schedule	68	3.99	0.63	66	4.47	0.44	6.84***	0.76
Positive learning climate	68	3.93	0.84	66	4.40	0.58	4.38***	0.56
Assessment/IEP development	68	3.43	0.83	66	4.13	0.58	7.70***	0.84
Curriculum and instruction	68	3.35	0.71	66	4.09	0.64	9.52***	1.04
Communication	68	2.47	0.96	66	3.50	0.98	6.80***	1.07
Social	68	3.00	0.81	66	3.88	0.87	8.16***	1.09
Staff/peer relationships	68	2.95	0.96	66	3.76	0.82	6.57***	0.84
Personal independence and competence	67	3.58	0.91	66	4.17	0.80	5.48***	0.65
Functional behavior	68	4.20	0.80	66	4.60	0.56	6.04***	0.50
Family involvement	68	3.80	0.69	66	4.38	0.51	7.08***	0.84

***p < .001

## Discussion

While there has been an increase in the number of children and youth with ASD served by the public schools and a need for determining the quality of services provided, there has not previously been a standardized, psychometrically validated instrument that assesses program quality for this group of children and youth. This study contributes to the literature by providing psychometric evidence for the reliability and validity of one such instrument—the *APERS*.

The first research question addressed the instrument reliability of the *APERS*. Analyses of internal consistency (Cronbach 1951, 1970) occurred for two data sets. Using guidelines for interpreting alphas established by Cicchetti (1994) and others (i.e., < .70 = poor; .70–.79 = fair; .80–.89 = good; ≥.90 = excellent), the overall internal consistency for the *APERS* was excellent, and alphas for the domain scores were primary in the fair to good range. The lower scores may have been in part due to the small number of items in some domains. Resource limitation prevented collecting inter-rater agreement data for the NPDC sample, but it was collected on the CSESA sample. The ICC and correlation between raters on mean item scores was moderate, but the mean item total *APERS* score for the two raters when averaged across programs were nearly identical. Even with this evidence for inter-rater agreement, it is important to specify that the *APERS* is a rating scale rather than a direct observational system in which event, interval-sampling, or momentary-time sampling data are collected. For the latter measures, exact agreement on whether a behavior did or did not occur is necessary (Yoder and Symons 2010). For the *APERS*, raters gathered information from observations, multiple interviews, and written documents, and used the convergence of information to make judgments about items ratings. For rating scales, measures of internal consistency are more important indicators of reliability (Henson 2001).

From our review of the literature and examination of other assessments of program quality, we proposed a conceptual framework of program quality that contained 10 subdomains. To provide evidence of construct validity, we conducted a confirmatory factor analysis that indicated the domains loaded on a single factor solution. We then replicated this finding with a second, independent set of data collected in high schools. As noted, the RMSEA score was higher than generally considered acceptable, and it is possible that RMSEA may have been affected by our relatively small sample size (Kenny et al. 2015). However, we propose the factor loadings provides stronger evidence for the construct validity of the *APERS*. These analyses provided support for our hypothesis that multiple features of programs for children and youth with ASD will contribute to an overall construct of program quality.

Another indicator of construct validity is the sensitivity of an instrument to changes consistent with a hypothesized effect (Hale and Astolfi 2014). In a study conducted with the NPDC sample, (Odom et al. 2013) analyzed changes in total *APERS* score for a group of school programs in which staff implemented a quality improvement plan (i.e., based on *APERS* pretest scores) in their schools. The *APERS* detected significant differences across time that were consistent with the researchers’ hypotheses. The next step in this research will be to examine use of the *APERS* in an experimental study in which a model, like NPDC or CSESA for example, is implemented in a set of schools and is compared to similar set of schools randomly assigned into a control condition to determine if it can detect treatment effects.

Several methodological limitations to this study exist. Although data were collected by a nationally distributed set of researchers, all were members of a single research group. Replication by independent researchers would be a desirable next step. Also, psychometric evaluations of instruments with 60+ items require a large number of participants, which in this case are classrooms or schools. For some analyses (e.g. factor analysis), we approached being “under-powered,” and our analyses would have benefited from having more participants (i.e., schools). In addition, for research involving children and youth with ASD, it is important to include information about the race and ethnicity of the sample (Pierce et al. 2014). This information was available for the CSESA sample but not for the NPDC sample.

Another limitation was that the programs involved in this study were a “convenience sample” (i.e., part of large professional development or experimental research projects). It would have been optimal to have had a stratified random selection of programs. However, when working in authentic settings such as schools in communities, addressing broad, important questions sometimes may come at the cost of control one could achieve with more narrowly defined samples (e.g., individual students vs. schools). Also, we noted that the sample is “small” for the type of psychometric analyses required of assessment instruments, but in comparison to other studies noted in the literature review, this study is the largest, to date, to directly assess program quality in school setting for students with ASD, although Renty & Roeyers (2005) did have a larger number of schools that responded to their survey research study in Belgium.

The conceptual framework proposed in Fig. 1 suggested that program quality may serve as a foundation for implementation of evidence-based practices. We proposed that program quality is similar to the concept of “common factors” clinical psychology therapeutic practice. This concept is new to the field of education. A direction for future research would be to examine empirically the relationship between program quality and the fidelity of implementation of EBPs in programs for children and youth with ASD.

The practical significance of this study is that it demonstrated reliability and validity of a measure of quality for school-based programs for children and youth with ASD. The data from these studies were collected in a geographically diverse set of school programs that were both inclusive and non-inclusive. The psychometric evidence underlying the *APERS* may allow practitioners and administrators to use with confidence the instrument for a variety of purposes. One purpose may be formative evaluation and program improvement. As noted in (reference blinded, to be included in final manuscript) and previously in this study, focused program development based on the APERS appears to improve program quality. This finding, however, would need to be confirmed in studies that employ randomized experimental design methodology.

Another practical implication is that the *APERS* may be a tool that could be used to communicate with families the quality of programs for their children with ASD. Often, issues of quality are points of heated discussion between school personnel and family members (Yell et al. 2003). Domains and indicators on the *APERS* may be beneficial to both parties in that they will allow discussion to focus more on specific practices and processes rather than generalized, ill-defined concepts of quality. In addition to the practical implications, the *APERS* now provides a psychometrically sound tool that may be used in research on educational programs for children and youth with ASD.

Along with the benefits there are some practical limitations. The *APERS* does require training. It is not an “off the shelf” product. Training of a single staff member who may then train others in a system takes 3–4 days. Also, the *APERS* assessment is itself labor intensive. The total staff time for one school (i.e., scheduling, observing, interviewing, reading records) has often required 2 days. It does, however, provide a comprehensive assessment of features of the program that are critically important for students with ASD.

In conclusion, this study provides support for the *APERS* as a measure of quality for school-based programs for children and youth with ASD. High quality programs establish the foundation for teachers’ implementation of evidence-based practices. The *APERS* should be viewed as a “starting place” that is necessary, but possibly not sufficient, for implementing instruction or intervention that links student goals to evidence-based focused intervention practices. The further steps of having measureable and observable goals and linking those goals to rigorously identified EBPs (see reference blinded, to be included in final manuscript) are essential in effective programs for children and youth with ASD.
